# Reducing Nitrogen Input in Barley Crops While Maintaining Yields Using an Engineered Biostimulant Derived From *Ascophyllum nodosum* to Enhance Nitrogen Use Efficiency

**DOI:** 10.3389/fpls.2021.664682

**Published:** 2021-05-05

**Authors:** Oscar Goñi, Łukasz Łangowski, Ewan Feeney, Patrick Quille, Shane O’Connell

**Affiliations:** ^1^Plant Biostimulant Group, Shannon Applied Biotechnology Centre, Munster Technological University-Tralee, Tralee, Ireland; ^2^Brandon Bioscience, Tralee, Ireland

**Keywords:** biostimulant, *Ascophyllum nodosum* extract, nitrogen use efficiency, barley, yield, sustainability, GHG emissions

## Abstract

Intensive agricultural production utilizes large amounts of nitrogen (N) mineral fertilizers that are applied to the soil to secure high crop yields. Unfortunately, up to 65% of this N fertilizer is not taken up by crops and is lost to the environment. To compensate these issues, growers usually apply more fertilizer than crops actually need, contributing significantly to N pollution and to GHG emissions. In order to combat the need for such large N inputs, a better understanding of nitrogen use efficiency (NUE) and agronomic solutions that increase NUE within crops is required. The application of biostimulants derived from extracts of the brown seaweed *Ascophyllum nodosum* has long been accepted by growers as a sustainable crop production input. However, little is known on how *Ascophyllum nodosum* extracts (ANEs) can influence mechanisms of N uptake and assimilation in crops to allow reduced N application. In this work, a significant increase in nitrate accumulation in *Arabidopsis thaliana* 6 days after applying the novel proprietary biostimulant PSI-362 was observed. Follow-up studies in barley crops revealed that PSI-362 increases NUE by 29.85–60.26% under 75% N input in multi-year field trials. When PSI-362 was incorporated as a coating to the granular N fertilizer calcium ammonium nitrate and applied to barley crop, a coordinated stimulation of N uptake and assimilation markers was observed. A key indicator of biostimulant performance was increased nitrate content in barley shoot tissue 22 days after N fertilizer application (+17.9–72.2%), that was associated with gene upregulation of root nitrate transporters (*NRT1.1*, *NRT2.1*, and *NRT1.5*). Simultaneously, PSI-362 coated fertilizer enhanced nitrate reductase and glutamine synthase activities, while higher content of free amino acids, soluble protein and photosynthetic pigments was measured. These biological changes at stem elongation stage were later translated into enhanced NUE traits in harvested grain. Overall, our results support the agronomic use of this engineered ANE that allowed a reduction in N fertilizer usage while maintaining or increasing crop yield. The data suggests that it can be part of the solution for the successful implementation of mitigation policies for water quality and GHG emissions from N fertilizer usage.

## Introduction

Nitrogen (N) is a critical nutrient to provide optimal growth and yield of all agricultural crops. The invention of the Haber–Bosch process for the production of N and its role in the green revolution during the nineteen sixties led to transformative improvements in crop yields, allowing sustained population growth worldwide. Large amounts of N fertilizer are currently intensively supplemented by growers every season in the form of nitrate (NO_3_^–^), ammonium (NH_4_^+^) or urea. The annual global demand for N chemical fertilizers is continuously increasing and is driven by population growth and a global shift toward a more protein-rich diet in developing countries (Lassaletta et al., 2016). Estimated global N containing fertilizer use in 2020 was 110 Mt (IFA, 2019). However, it is also estimated that the energy demanding Haber–Bosch process for the production of N chemical fertilizers consumes 2% of the world’s energy supply as fossil fuels, making N fertilizer expensive to produce and representing a relevant cost source for growers (Pfromm, 2017).

Nitrate is the predominant form of N taken up by plants from the soil due to its negative charge and high soil mobility, however most plants also benefit from inclusion of N in the form of ammonium in order to boost their N content (Hachiya and Sakakibara, 2017; Vidal et al., 2020). Crop plants are intrinsically inefficient at accessing applied N. Depending on the crop, agronomy practices and soil type, more than half of the supplemented N fertilizer can be lost to the environment (Raun and Johnson, 1999; White and Brown, 2010; Gutiérrez, 2012; Yan et al., 2020). These losses are not only increasing the cost of production but most importantly are increasing environmental pollution. Nitrogen fertilizers that are not retained in the soil, are washed out and leached to waterways and into groundwater. Nitrogen is an important contributor to eutrophication and water quality (Ascott et al., 2017; Wang et al., 2019). Furthermore, high amounts of greenhouse gas (GHG) emissions are associated with N use, either directly through N fertilizer production (mainly CO_2_ and N_2_O) or indirectly via emission of nitrous oxide gas (N_2_O) by denitrification processes in the soil (Schaufler et al., 2010). Therefore, maintaining high crop yields while decreasing N fertilizer requirements by improving N use efficiency (NUE) in crops is essential to produce sustainable and environment friendly food. The potential economic benefits to growers of using N more efficiently is compelling, an improvement of NUE in crops by 1% could save approximately $1.1 billion annually (Kant et al., 2011; Kanter et al., 2015).

As a phenotypic trait, NUE is frequently defined by crop breeders/plant scientists as the rate of conversion of N input (e.g., N- fertilizer applied and/or soil N) into total plant biomass or specific plant organ biomass (e.g., grain yield). NUE is an inherently complex trait because it involves interaction between genetic, environmental and agronomy factors. NUE can be broken down into two different components for a crop: N uptake efficiency (Nupeff) and N utilization efficiency (Nuteff) (Moll et al., 1982; Anbessa and Juskiw, 2012; Hawkesford and Riche, 2020). Nupeff is the ratio of N taken up by the crop compared to what N is available from the soil and/or applied as fertilizer. Nuteff can be calculated as the amount of crop produced per unit of N taken up by the crop. The plant biological processes responsible for NUE include: N uptake, N transport, N assimilation, N translocation and recycling when the plant or its organs are aging. Each step is tightly controlled at gene transcriptional, translational, and post-translational level and includes low and high-affinity N transporters (Noguero and Lacombe, 2016; Hachiya and Sakakibara, 2017; Wang et al., 2018; Vidal et al., 2020) along with enzymes related to N assimilation pathways such as nitrate reductase (NR), glutamate synthase (GOGAT), and glutamine synthetase (GS) (Tischner, 2000; Masclaux-Daubresse et al., 2010; Pratelli and Pilot, 2014; Hirel and Krapp, 2020).

Approaches for increasing NUE for crops includes selective plant breeding, genetic modification, soil management and fertilizer technologies/management. Breeding programs to obtain high grain yielding varieties growing under contrasting/reduced N regimes have focused on traits such as bigger root length or surface area, larger above-ground biomass, higher grain harvest index or enhanced photosynthetic capacity (Anbessa and Juskiw, 2012; Prey et al., 2019; Vicente et al., 2019; Voss-Fels et al., 2019). Genetic modification techniques have produced new varieties of some crops able to increase biomass and seed yield under reduced N inputs through manipulation of genes for N uptake and transport (Fang et al., 2013; Han et al., 2016; Wang et al., 2018; Li et al., 2020), N assimilation (Perchlik and Tegeder, 2017; James et al., 2018; Gao et al., 2019) and N remobilization (Chen et al., 2020). However, it is important to note that these new varieties have been mainly developed in pot or hydroponic systems and have not been validated in field conditions (Masclaux-Daubresse et al., 2010; Wang et al., 2018). The potential impact of novel fertilizer technologies such as urease and nitrification inhibitors or slow-release fertilizer coatings to enhance NUE and/or mitigate GHG emissions have been intensively studied over the last years, showing promising results. However, specific NUE data is limited and should be interpreted cautiously as only a few studies included reduced N rates in the trial designs (Snyder, 2017; Rose et al., 2018; Dimkpa et al., 2020).

The new EU Fertilising Products Regulation, which entered into force on 15 July 2019, establishes plant biostimulants as a separate category of fertilizers that are defined by their ability to improve nutrient use efficiency, tolerance to abiotic stress and/or quality traits regardless of their nutrient content (EU Regulation 2019/1009, 2019). Plant biostimulants have gained significant attention from scientists, the agroindustry, and growers over recent years as more sustainable solutions for crop production are sought (Du Jardin, 2015; Rouphael and Colla, 2020). Globally, the biostimulant market accounted for $3.19 billion in 2019 and is expected to reach $9.22 billion by 2027, growing at a CAGR of 14.2% during the forecast period (Research and Markets, 2020). Biostimulants made using extracts from the brown seaweed *Ascophyllum nodosum* (ANE) are accepted as effective and robust products (Yakhin et al., 2017; Shukla et al., 2019). A number of publications have demonstrated that ANE biostimulants improve fruit quality (Frioni et al., 2018), reduce pod shattering (Łangowski et al., 2019), enhance tolerance to abiotic stresses (Goñi et al., 2018; Carmody et al., 2020) and improve nutrient uptake in diverse crops (Turan and Köse, 2004; Jannin et al., 2013; Billard et al., 2014; Sabir, 2014; Stamatiadis et al., 2015; Frioni et al., 2018; Laurent et al., 2020). However, little is known on how ANEs can influence mechanisms of N uptake and assimilation in crops under reduced N conditions. The integration of biostimulants with current crop husbandry practices to enhance NUE may present challenges (i.e., method of application, timing, rates, etc.) in some production systems. These challenges can be overcome with a consistent and positive yield response trend in field conditions, economic/environmental justification for their use and acceptance by relevant stakeholders of the supporting scientific evidence on the effectiveness of the technology.

In this study, we investigated the potential of an ANE based biostimulant, PSI-362, to increase NUE in barley crops under reduced N input in multi year field trials. The method of application of the biostimulant as a foliar spray or top dressing was also investigated. In order to assess the impact on NUE, well-established N uptake and assimilation plant parameters at phenotypical, metabolic, enzymatic, and molecular level were evaluated in the model plant *Arabidopsis thaliana* and barley crop. Overall, the results support the agronomic use of this engineered ANE that allowed a 25% reduction in N fertilizer usage while maintaining or increasing crop yield.

## Materials and Methods

### Materials

ANE biostimulant containing the PSI-362 biomolecule complex was provided by Brandon Bioscience (Tralee, Ireland). The compositional characterization of PSI-362 was performed according to Carmody et al. (2020) and consisted of ash (43.7% w/w dry), carbohydrates (26.0% w/w dry), polyphenols (12.3% w/w dry) and other organic components (18% w/w dry). The N:P:K macronutrient content of PSI-362 was 0.4:0.1:8.0% w/v. Commercially available granular CAN + S (27% w/w N; 4% w/w S) was kindly provided by Target Fertilisers (Enniscorthy, Ireland). CAN + S was coated with PSI-362 biomolecule complex using standard fertilizer coating technology. All chemical reagents used for the biochemical assays were purchased from Sigma-Aldrich (Arklow, Ireland) and Bio-Rad (Watford, United Kingdom). Phytostrip tubes were purchased from 4titude Ltd. (Wotton, United Kingdom). The primers were purchased from Eurofins Genomics (Ebersberg, Germany).

### Evaluation of PSI-362 in *Arabidopsis thaliana* Seedlings

An initial evaluation of the ability of PSI-362 to improve NUE and nitrate content was carried out using *Arabidopsis thaliana* (Col-0) seedlings. This experiment was based on a high throughput root microphenotyping platform published previously (Vasilieva et al., 2021). Arabidopsis seeds were sterilized and grown on Phytostrip tubes filled with a sterile solid medium and inserted into 96-well plates (six seeds per tube). This medium contained Phytagel, sucrose, ammonium nitrate as N source, magnesium chloride and calcium chloride. Once Arabidopsis seeds germinated, a liquid nutrient medium based on Gamborg’s B-5 (B5) medium was added to the wells. Seedlings were subjected to two different N conditions by the selective addition of ammonium nitrate to this liquid medium (2.66 and 13.33 mM, respectively). PSI-362 was applied as biostimulant treatment to the same liquid medium under low and high N conditions at 0.04% w/v per well. Distilled water was applied as an untreated control. Arabidopsis seedlings grew for 6 days at a temperature of 22/21°C (day/night; 16/8 h) and 70% relative humidity (RH) under a light intensity of 100 μmol⋅m^–2^⋅s^–1^. After this time period, Arabidopsis seedling tissue was collected to determine nitrate content as described in Section “Measurement of N Metabolites, Free Amino Acids, and Soluble Protein.”

### Experimental Design of Barley Pot Trial

The pot experiments were performed in a cultivation room under controlled conditions (19/14°C with 16 h of light and 8 h of darkness and 80 ± 5% RH under a light intensity of 120 μmol⋅m ^–2^⋅s^–1^). Winter barley plants (cv. Towers; Supplementary Table 1) were grown in pots with a capacity of 2 L filled with a model soil prepared by mixing one part of sand and two parts of coarse loamy soil from field trials area described in Section “Experimental Design of Field Trials With PSI-362 Applied as Foliar Spray” (bulk density 1.41 g⋅cm^–3^, pH 6.1, 33.60 g⋅kg^–1^ DW organic matter, 2.50 g⋅kg^–1^ DW total N, 230.77 g⋅kg^–1^ amino sugar-N, 2.59 mg⋅kg^–1^ DW available P, and 63.36 mg⋅kg^–1^ DW available K). Soil N, P, and K index was defined as 1, 1, and 2, respectively (Teagasc, 2015). Experiments were performed in three independent pots per treatment, with 20 plants per pot. Barley seeds were sown at a depth of 3 cm and plants were irrigated with 1 liter of water per pot every 3 days in order to create equal soil moisture conditions in all pots. Temperature and relative moisture content were recorded regularly with a portable USB data logger (EBI300 TH, Ebro Electronic). The experiment was completed for three different treatments: (1) untreated control fertilized with CAN + S; (2) treated with PSI-362 foliar spray and fertilized with CAN + S; (3) treated with CAN coated with PSI-362. Granular N fertilizer addition (CAN or coated CAN with PSI-362) was added to 7-day-old plants (growth stage GS12-14) on the soil surface at a rate of 67 kg N⋅ha^–1^ (75% of recommended N at this growth stage). Treated plants with foliar spray received the same amount of PSI-362 as those that were treated with the coated fertilizer. The barley plants were harvested 14 days after receiving treatments (growth stage GS22-23). The root and the above ground parts of the plants were washed and dried at 100°C for 24 h to determine both fresh weight (FW) and dry weight (DW). The efficiency of the conversion of added inorganic N into total plant dry matter (DM) was calculated per independent pot:

$${\mathrm{NUE}}_{\mathrm{plant}}={\mathrm{DM}}_{\mathrm{plant}}/N_{\mathrm{fertilized}}$$

### Experimental Design of Field Trials With PSI-362 Applied as Foliar Spray

We initially investigated the effect of PSI-362 applied by foliar spray on grain yield and NUE in recommended spring and winter barley varieties (Supplementary Table 1) growing under reduced N fertilizer rate (75%) and compared to standard grower practice (100% N). These open-field experiments were conducted in Tralee, Co. Kerry, Ireland (52° 16′ N, 9° 41′ W and 16 m altitude) during the 2016, 2017, and 2018 growing seasons. The climate is classified as temperate oceanic (Cfb) according the Köppen climate classification. The average minimum, maximum and mean temperatures of 9.9°C, 18.2°C, and 14.1°C, and a mean monthly accumulated precipitation of 63.0 mm was recorded during the field trial period. The soil type was coarse loamy, and the previous crop grown in the field trial site was grass. Soil characteristics were evaluated every season and representative samples were collected in a W shaped pattern across the sampling area. Soil bulk density was measured with a volumetric cylinder, soil pH was measured in buffer pH 4–10 to also estimate the lime requirements, organic matter content was determined gravimetrically after burning the sample at 440°C for 12 h, total N evaluation was carried out using the Kjeldahl method, amino sugar quantification was performed using the Illinois test, and available K and P were extracted through the Morgan method and quantified by ICP-MS and spectrophotometry, respectively. The obtained values for these trials were: bulk density 1.22 g⋅cm^–3^, pH 5.6–6.1, 43.10–49.50 g⋅kg^–1^ DW organic matter, 3.40–4.10 g⋅kg^–1^ DW total N, 305–388 g⋅kg^–1^ amino sugar-N, 2.98–3.92 mg⋅kg^–1^ DW available P, and 78.20–92.50 mg⋅kg^–1^ DW available K. Soil N, P, and K index was defined as 1, 2, and 2, respectively (Teagasc, 2015). Every year, the field trials area was rotated to overcome any carryover effects from the previous year.

Two spring and one winter barley varieties (cv. KWS Irina, cv. Mickle, and cv. KWS Towers; Supplementary Table 1) were sown by mechanic drilling at a seed rate of 180–190 and 150–160 kg⋅ha^–1^ between March-April and October, respectively. The experimental design was the same for all field trials sites: a randomized complete block with five replicates and experimental units (plots) measuring 3 m × 5 m (15 m^2^). N fertilization was designed to follow conventional farming practices in Ireland according to the Agriculture and Food Development Authority (Teagasc) recommendations (Teagasc, 2015). A quantity of 155 and 117 kg N⋅ha^–1^ was supplied in two applications for spring barley growing under 100% N rate and 75% N rate, respectively. 200 and 150 kg N⋅ha^–1^ was supplied in four applications for winter barley growing under 100% and 75% N rate, respectively. Barley crops were treated three times by foliar spray with PSI-362 (1 L⋅ha^–1^) at mid tillering stage (GS22-27), at early stem elongation stage (GS30-31), and at the start of the booting stage (GS41-43). Water was sprayed as a control in the untreated plants. A central 1 m^2^ area from the plots was harvested manually at barley maturity (GS92) and processed by a cereal threshing machine. Grain yield was measured for each plot and corrected to a standard moisture of 20%. Additional information of the grower program for spring and winter barley trials can be found in Supplementary Tables 2, 3, respectively.

### Experimental Design of Field Trials With PSI-362 Coated Fertilizer

In the 2019 growing season, the effect of PSI-362 coated CAN + S fertilizer on spring barley (cv. Gangway) was evaluated in two independent open-field trials under different reduced N fertilizer rates (73% and 88%) and compared to standard grower practice (100% N). Field 1 was laid out in Ardfert, Co. Kerry, Ireland (52° 22′ N, 9° 45′ W and 16 m altitude) from April 2 to August 28, 2019. Field 2 was conducted in Courtnacuddy, Co. Wexford, Ireland (52° 28′ N, 6° 42′ W and 82 m altitude) from April 1 to August 19, 2019. The climate for both locations is classified as temperate oceanic (Cfb) according the Köppen climate classification. The average minimum, maximum, and mean temperatures of 7.9°C, 15.4°C, and 11.7°C, and a mean monthly accumulated precipitation of 78.21 mm was recorded during the field 1 trial period. The field 2 trial period was characterized by an average minimum, maximum and mean temperatures of 6.7°C, 14.6°C, and 10.8°C, and a mean monthly accumulated precipitation of 90.73 mm. The field 1 soil type was coarse loamy, and the previous crop rotation was potato. The field 2 soil type was clay loam, and spring barley was grown in the previous rotation. Soil chemical characteristics for both fields were evaluated before sowing and after harvesting according to the same methodology described in Section “Experimental Design of Barley Pot Trial” (Supplementary Tables 4, 5). For both fields, soil N, P, and K index was defined as 1, 4, and 4, respectively (Teagasc, 2015).

Barley was sown at the beginning of April by mechanical drilling at a seed rate of 190 and 140 kg⋅ha^–1^ for field 1 and 2, respectively. Split plot design was used for both field trials and the average area of each condition (control 100% N or PSI-362 treated reduced N fertilizer) was 1.25 and 6.10 ha for field 1 and field 2, respectively. A quantity of 140.6 and 102.8 kg N⋅ha^–1^ was supplied in two applications for field 1 under untreated 100% N rate and PSI-362 treated 73% N rate, respectively. A common first application of NPK 10-10-20 (380 kg⋅ha^–1^) was added first to both conditions on sowing. A second application with CAN + S (380 kg⋅ha^–1^) and PSI-362 coated CAN + S (240 kg⋅ha^–1^) was applied on untreated and treated plants at mid tillering stage (GS22-27), respectively. Regarding field 2, 158.1 and 140.5 kg N⋅ha^–1^ was supplied in two applications for untreated 100% N rate and PSI-362 treated 88% N rate, respectively. An initial application of NPK 22-8-0 (375 kg⋅ha^–1^) added in the seedbed to both conditions was followed by a second application with CAN + S (280 kg⋅ha^–1^) and PSI-362 coated CAN + S (215 kg⋅ha^–1^) to untreated and treated plants at mid tillering stage (GS22-27), respectively. Additional information of the grower program for both trials can be found in Supplementary Table 6.

Shoot and root tissue from untreated and treated fields were sampled and phenotypically evaluated 22 days after the second fertilizer application in plants at early stem elongation stage (GS30-31). The samples were snap-frozen in liquid nitrogen, ground and kept in −80°C until further biochemical, enzymatic and gene expression analysis. Field 1 was manually harvested at growth stage GS92 using at least six independent 1 m^2^ plots and grain was processed through a cereal trashing machine. Field 2 was harvested at the same maturity stage using a combine harvester. Before grain harvest from each plot, whole plant samples were collected for further phenotypical determination.

### Plant and Grain Measurements of Field Trials With PSI-362 Coated Fertilizer

Barley plants were harvested at 22 days after second fertilizer application (stage GS30-31) and at grain maturity (GS92). The number of plant tillers was also counted. Plants at early stem elongation stage (GS30-31) were divided into roots and shoots and FW was measured. Plant DW was determined by drying these fresh samples in a convection oven for 24 h at 100°C. Plants harvested at maturity stage (GS92) were divided into roots, grains, remaining ears (glumes and beards), and remaining shoots (stems and leaves) and dried for 72 h at 50°C before biomass determination. Obtained values were expressed at 20% moisture. The harvest index was calculated as the grain biomass divided by the above-ground plant biomass. Grain yield was determined from the harvested and trashed plots, corrected to a to a standard moisture of 20%, and extrapolated t⋅ha^–1^. A random grain subsample from each plot was used to determine the thousand-grain weight (TGW). N content was determined in grain samples at 20% moisture through the Kjeldahl method and grain protein content was calculated by multiplying the N content by the barley-specific protein factor of 5.83 (Mariotti et al., 2008). Total P content was determined from the same grain digest samples of the Kjeldhal method through the molybdovanadate method. Total K content was determined in grain after nitric acid extraction and further evaluation by atomic emission spectroscopy.

### Calculation of NUE and Its Components for Field Trials

*NUE*_*grain*_ was calculated as the yield of grain produced per unit of N fertilizer and expressed as kg grain per kg N fertilizer:

$${\mathrm{NUE}}_{\mathrm{grain}}=\mathrm{Grain yield}/N_{\mathrm{fertilized}}$$

Grain N uptake efficiency (*Nupeff*_*grain*_) was calculated as the grain N uptake (*Nup*_*grain*_) in relation to the quantity of fertilizer and expressed as kg N available in grain per kg N fertilizer. *Nup*_*grain*_ was calculated by multiplying its nitrogen content (*N%*) by grain yield at 20% moisture:

$${\mathrm{Nupeff}}_{\mathrm{grain}}={\mathrm{Nup}}_{\mathrm{grain}}/N_{\mathrm{fertilized}}=(\mathrm{Grain yield}\times {\left(N\%\right)}_{\mathrm{grain}})/N_{\mathrm{fertilized}}$$

Grain N utilization efficiency (*Nuteff*_*grain*_) was calculated as the amount of grain produced per *Nup*_*grain*_ and expressed as kg grain per kg N available in grain:

$${\mathrm{Nupeff}}_{\mathrm{grain}}=\mathrm{Grain yield}/{\mathrm{Nup}}_{\mathrm{grain}}=\mathrm{Grain yield}/(\mathrm{Grain yield}\times {\left(N\%\right)}_{\mathrm{grain}})$$

The soil N component was not considered for the calculation of NUE traits as per the methodology suggested by Prey et al. (2019).

Greenhouse gas emissions from field trials with PSI-362 coated fertilizer were estimated using a carbon footprint decision support tool for spring barley (AHDB, 2012) and expressed as kilograms of carbon dioxide equivalent (kgCO_2_e) per hectare. The information required to calculate this carbon footprint was taken from field trial records (crop and soil type, grain yield and moisture, fertilizer rate, crop protection inputs, crop residue management, field energy use, and grain drying). PSI-362 manufacture carbon footprint was also included in the estimation for treated crops.

### Measurement of N Metabolites, Free Amino Acids, and Soluble Protein

Nitrate content was determined in Arabidopsis seedlings and barley shoot tissue according to nitration of salicylic acid under acidic conditions as described by Hachiya and Okamoto (2017) and quantified as mg⋅g^–1^ DW. Free amino acids and ammonium extraction was performed by mixing 40 mg of barley shoot tissue with 800 μl of ethanol 70% (v/v), kept overnight at 4°C under dark and centrifuged at 20,000 × *g* for 10 min at 4°C. These supernatants were first used for the estimation of total free amino acids content using ninhydrin reagent. 100 μl of supernatant was mixed with 75 μl of reaction mixture [8% (w/v) ninhydrin in acetone] and incubated for 30 min at 80°C. After cooling at room temperature, 100 μl of ethanol 50% (v/v) was added and the absorbance was measured at 570 nm. After preparing a calibration standard curve with glutamic acid, total free amino acids content was expressed as mg⋅g^–1^ DW. Individual free amino acids and ammonium content was evaluated in the same extracts through a reversed phase high performance liquid chromatography (RP-HPLC) and UV detection at 280 and 300 nm of the aminoenones formed by the reaction of amino acids and ammonia with the derivatization reagent diethyl ethoxymethylenemalonate (DEEMM) (Gómez-Alonso et al., 2007). Compounds were identified by comparison of retention time to that of commercial standards, quantified by peak integration and expressed as mg⋅g^–1^ DW. Soluble protein content was measured from the extracts used for the NR and GS enzymatic assays (see section “NR and GS Activity Assays”) through the modified version of the Bradford method in a 96 well plate using a protein-dye reagent (Bio-Rad) and expressed as mg⋅g^–1^ DW.

### Measurement of Photosynthetic Pigments

Photosynthetic pigments from collected barley shoot tissue (chlorophyll a, chlorophyll b and carotenoids) were extracted by homogenizing 40 mg of shoot tissue with 1 ml of acetone 80% (v/v). After incubation for 5 h at 4°C, samples were centrifuged at 20,000 × *g* for 10 min at 4°C. The supernatants were collected and diluted with acetone 80% (v/v) before measuring the absorbance at 470, 647 and 663 nm. Empirical equations described by Lichtenthaler and Buschmann (2001) for total chlorophylls (chlorophyll a + chlorophyll b) and carotenoids concentrations were used and results were expressed as mg⋅g^–1^ DW.

### NR and GS Activity Assays

For the extraction of NR and GS, frozen ground barley shoot tissue was homogenized in the extraction buffer [50 mM HEPES-KOH (pH 7.5), 10% glycerol (v/v), 2% (w/v) PVPP, 2 mM DTT, 1/300 dilution protease inhibitor cocktail P9599 (Sigma-Aldrich)] for 2 h at 4°C, followed by centrifugation at 20,000 × *g* for 20 min at 4°C. NR activity was measured in the obtained protein extracts in the absence of MgCl_2_ (total activity) and in the presence of MgCl_2_ (actual activity). The reaction was incubated for 45 min at 30°C after the addition of 60 μl of protein extract to 340 μl of reaction buffer containing 50 mM HEPES-KOH (pH 7.5), 10 mM KNO_3_, 5 mM EDTA (total activity) or 5 mM MgCl_2_ (actual activity), 10 mM FAD, 1 mM DTT, and 0.2 mM NADH. The reaction was stopped by adding 120 μl of 0.6 M zinc acetate and the samples were centrifuged at 25,000 × *g* for 5 min. The amount of produced nitrite was determined spectrophotometrically at 540 nm according Hachiya and Okamoto (2017) and NR activity was expressed as nmol NO_2_^–^⋅h^–1^⋅mg^–1^ protein. The activation state of NR was defined as the activity measured in the presence of 5 mM MgCl_2_ divided by the activity measured in the presence of 5 mM EDTA (expressed as a percentage). GS was determined by incubating 60 μl of protein extract with 120 μl of reaction buffer (50 mM Tris-HCl (pH 7.5), 20 mM MgSO_4_, 4 mM EDTA, 80 mM sodium glutamate, 6 mM hydroxylamine, and 8 mM ATP) for 60 min at 30°C. The reaction was stopped by adding 180 μl of 0.122 M FeCl_3_, 0.5 M TCA, and 2 M HCl and the samples were centrifuged at 16,000 × *g* for 10 min at 4°C. The absorbance of γ-glutamylmonohydroxamate (γ-GHM) in the supernatant was measured at 560 nm, with γ-GHM used as the standard for the calibration curve. GS activity was expressed as μmol γ-GHM⋅h^–1^⋅mg^–1^ protein.

### RNA Extraction and RT-qPCR

Total RNA was isolated from frozen ground barley root tissue harvested in field 1 by Plant/Fungi Total RNA Purification Kit (Norgen Biotek, Canada) following the manufacturer’s instructions. RNA was treated with RNase-Free DNase I Kit (Norgen Biotek, Canada) in order to remove efficiently genomic DNA contamination. RNA concentration and purity was measured using Qubit (Thermo Fisher Scientific). Expression analysis of *HvNRT1.1* (*HORVU7Hr1G071600*), *HvNRT2.1* (*HORVU6Hr1G005590.1*), and *HvNRT1.5* (*HORVU6Hr1G070450.3*) genes was performed by RT-qPCR using a Roche LightCycler^®^ 96 System (Roche, United Kingdom) and a LightCycler^®^ RNA Master SYBR Green I one-step kit (Roche, United Kingdom) according to the manufacturer’s instructions. The expression level of the barley *HvUBC9* (*HORVU5Hr1G088270*) gene was used as the reference. The 2^–ΔΔ*CT*^ method was used to quantify relative normalized gene expression levels. The primers sequences used are shown in Supplementary Table 7.

### Statistical Analysis

Nitrate assessment in Arabidopsis seedlings was performed in at least four independent biological replicates per treatment and N condition (12 technical replicates per biological replicate). Phenotypic and NUE assessment of the barley pot trial was done in three independent pots, with 20 plants per pot. Grain yield, TGW, grain N content measurement, calculation of NUE derived traits, and GHG emissions was done in at least four independent biological replicates per treatment and variety. Soil characterization was performed in three independent replicates. Phenotype assessment in barley plants from field trials was carried out in at least 60 independent biological replicates. Shoot samples collected from barley plants at stage GS30-32 were pooled for further analysis. For biochemical and enzymatic analysis, at least four biological replicates of each treatment were performed, using three technical replicates per biological replicate. For gene expression analysis, at least three biological replicates of each treatment were performed, using three technical replicates per biological replicate. Statistics were evaluated with Sigma Plot 12 and Statgraphics Centurion XVI software. The differences between control and PSI-362 treatment for every field trial were analyzed using *t*-test at *p* ≤ 0.05. The effect of different treatments on the barley pot trial was analyzed with the one-way analysis of variance (ANOVA) by Tukey’s HSD test at *p* ≤ 0.05. Arabidopsis nitrate data were compared by using two-way ANOVA, with Tukey’s HSD test at *p* ≤ 0.05. The application of all parametric tests was performed after checking the data normality (Shapiro–Wilk’s test) and equal variance assumptions. Unless stated otherwise, all data are expressed as average ± standard error (SE). Details of the individual sample size for each analysis and statistical test used is mentioned in the tables and figure legends.

## Results

### Effect of PSI-362 in Nitrate Content of Arabidopsis Seedlings Under Different N Levels

**FIGURE 1:**
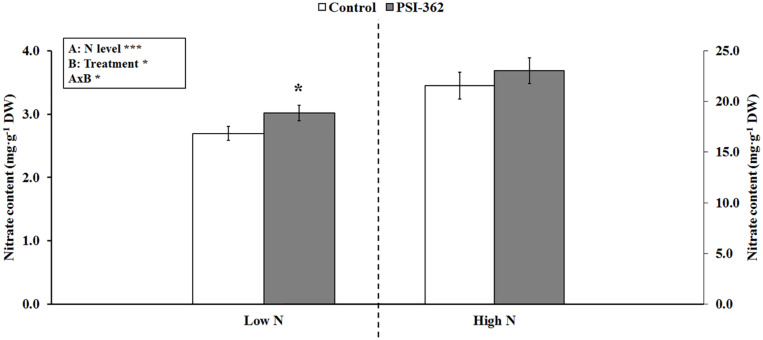
Nitrate content in Arabidopsis seedlings. The effect of PSI-362 added by root application to Arabidopsis seedlings growing under low and high N levels (2.66 and 13.33 mM ammonium nitrate, respectively) was evaluated 6 days after application. Since interaction AxB was significant (***p ≤ 0.001), data were subjected to *t*-test, comparing PSI-362 treatment versus control within the same N level. In this case, means followed by asterisk indicate statistically significant differences between control and PSI-362 treatment within the same N level (*p ≤ 0.05). Number of biological replicates (*n* ≥ 4).

The effect of PSI-362 was first evaluated in seedlings of the model plant *Arabidopsis thaliana* using a high throughput root microphenotyping platform. When a two-way ANOVA test was run, it was found that all three parameters (N level, PSI-362 treatment and N level × PSI-362 treatment) were statistically significant for nitrate content (Figure 1). Therefore, all data were subjected to *t*-test, comparing PSI-362 versus control effect under low and high N levels (2.66 and 13.33 mM of ammonium nitrate, respectively). While the different N amount added to untreated seedlings had a remarkable effect on nitrate content (low N: 2.69 mg⋅g^–1^ DW vs. high N: 26.46 mg⋅g^–1^ DW), our results also showed that PSI-362 applied through the root significantly increased this parameter by 11.92% (*p* = 0.049) and 6.85% (*p* = 0.420) compared to the low and high N control, respectively (Figure 1). It would suggest that PSI-362 can improve capacity for nitrate uptake in Arabidopsis under controlled conditions in the N range tested, but with the best performance at the lowest N rate.

### Effect of PSI-362 Mode of Application on Pot Experiments in Barley

**FIGURE 2:**
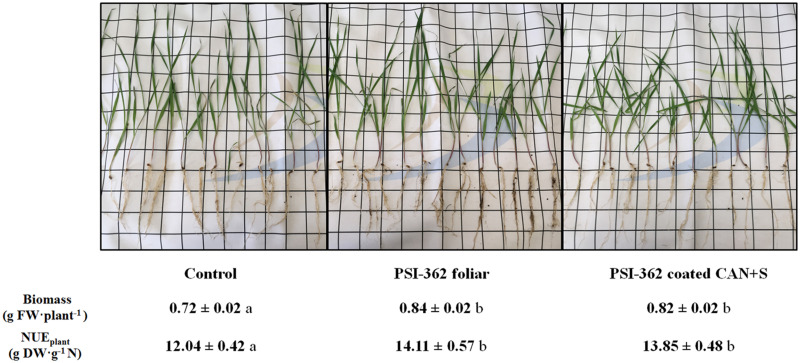
Effect of PSI-362 treatment applied by foliar spray or coated with CAN + S fertilizer on plant biomass and NUE of winter barley (cv. Towers). A pot trial was performed to evaluate if the application mode of PSI-362 (foliar spray or coated with N mineral fertilizer) affected differently to barley growth and compared with an untreated control. Fertilizer (CAN + S) and PSI-362 biostimulant were added to plants at 2–3 leaf stage (GS12–13) at the same time. The dose of PSI-362 applied by foliar spray or coating was exactly the same and control plants were sprayed with distilled water. The phenotypical assessment was performed 14 days after fertilizer/PSI-362 treatment application. Means followed by different small letter within the same row indicate significant differences between treatments based on one-way ANOVA Tukey’s HSD test at *p* ≤ 0.05. Number of biological replicates (plant biomass, *n* = 60; NUE, *n* = 3).

Granular CAN + S fertilizer was coated with PSI-362 and its biostimulant effect on plant biomass and *NUE*_*plant*_ was compared with the same amount of PSI-362 applied by a single foliar spray in a winter barley pot trial. As can be observed in Figure 2, regardless of the method of application, both PSI-362 treatments significantly increased plant biomass between 13.88 and 16.67% compared to untreated plants fertilized with the same amount of N (*p* ≤ 0.05). The measured phenotypic change also demonstrated a superior *NUE*_*plant*_ for barley plants treated with either PSI-362 coated fertilizer (+15.03%) or foliar PSI-362 (+17.19%), indicating that bioactive molecules from PSI-362 can be easily released from CAN + S granules and stimulate the plants through their root system to utilize N fertilizer more efficiently.

### Effect of PSI-362 Applied by Foliar Spray on Barley Yield and NUE

**FIGURE 3:**
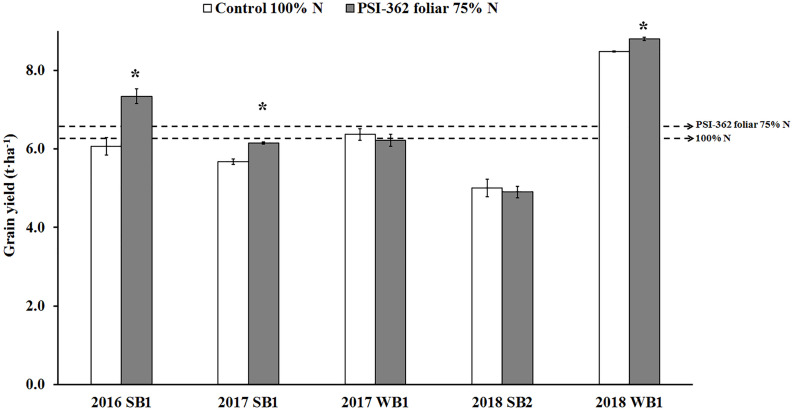
Effect of foliar PSI-362 treatment on grain yield of spring and winter barley varieties growing under 75% N fertilizer rate in 3 consecutive seasons (2016–2018). PSI-362 was applied three times by foliar spray at mid tillering stage (GS22-27), early stem elongation stage (GS30-31), and at the start of the booting stage (GS41-43), and its performance was benchmarked against untreated plants growing under 100% N fertilizer rate. SB1, spring barely cv. KWS Irina; WB1, winter barley cv. KWS Towers; SB2, spring barley cv. Mickle. Chart represents grain yield (t⋅ha^–1^) expressed at 20% moisture. Means followed by asterisk indicate statistically significant differences between control and PSI-362 treatment within the same field trial based on *t*-test at *p* ≤ 0.05. The horizontal dashed lines represent the average yield value for untreated 100% N (6.24 t⋅ha^–1^) and foliar PSI-362 75% N (6.57 t⋅ha^–1^). Number of plots harvested per field trial (*n* ≥ 5).

Three 1L/Ha foliar applications of PSI-362 (at tillering, stem elongation and booting stage) on recommended spring and winter barley varieties growing under reduced N fertilizer rate (75%) resulted in no difference or significantly higher grain yield for 5 out 5 field trials performed during 2016 to 2018 compared to plants growing under the recommended rate in standard agricultural practice (*p* ≤ 0.05) (Figure 3). Overall, PSI-362 treated barley varieties showed a statistically significant 3-year average yield increase of 5.57% (*p* = 0.011) using 25% less N. Similarly, the *NUE*_*grain*_ of the PSI-362 treatments was 29.85–60.26% higher than those of the control conditions (*p* ≤ 0.05) (Supplementary Table 8). The data demonstrates the ability of PSI-362 to deliver yield with a 25% reduction of N mineral fertilizer, significantly enhancing the amount of barley grain per unit of N supplied under field conditions.

### Effect of PSI-362 Coated Fertilizer on Vegetative Parameters at Early Stem Elongation Stage

In order to explore the effect of PSI-362 coated fertilizer on vegetative traits in spring barley, a comparative phenotypical analysis was performed on plants harvested at 22 days after the second fertilizer application (stage GS30-31). This analysis was performed on two independent field trials characterized by a different reduction of N fertilizer rate. Barley plants treated with PSI-362 coated fertilizer from field 1 (73% N) increased their shoot FW, root FW, root DW and number of tillers per plant by 6.91, 10.79, 21.05% and 9.45% compared to 100% N control, but these changes were not statistically significant (*p* > 0.05). While the effect of PSI-362 coated fertilizer in field 2 (88% N) was not statistically significant either, the mean values of shoot DW, root FW and root DW showed a similar increasing trend compared to the untreated plants (+14.11, +6.56, and +16.43%, respectively) (Table 1).

**TABLE 1:** Effect of PSI-362 coated CAN + S fertilizer on phenotypic parameters in barley crop (cv. Gangway) 22 days after application.

Parameter^1^	Field 1		Field 2	
	Control 100% N	PSI-362 coated 73% N	Control 100% N	PSI-362 coated 88% N
Shoot FW (g plant^–1^)	5.26 ± 0.26	5.62 ± 0.76	5.74 ± 0.32	6.55 ± 0.08
Shoot DW (g plant^–1^)	0.57 ± 0.03	0.54 ± 0.07	0.54 ± 0.02	0.57 ± 0.01
Root FW (g plant^–1^)	0.64 ± 0.06	0.71 ± 0.10	0.41 ± 0.02	0.43 ± 0.02
Root DW (g plant^–1^)	0.13 ± 0.01	0.16 ± 0.02	0.08 ± 0.01	0.10 ± 0.01
Number tillers per plant	4.23 ± 0.47	4.63 ± 0.68	4.83 ± 0.16	4.84 ± 0.24

*^1^Data are the means ± SE. Number of biological replicates (n = 30). No statistically significant differences between control and treated sample at p ≤ 0.05 were observed (t-test).*

### Effect of PSI-362 Coated Fertilizer on N Uptake, Metabolic and Molecular Parameters

**FIGURE 4:**
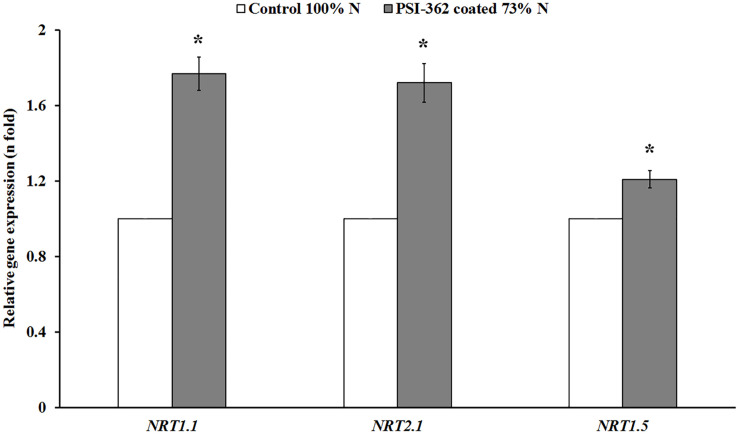
Relative expression of major nitrate transporter genes in spring barley root tissue (cv. Gangway). The effect of PSI-362 coated CAN + S fertilizer on the relative expression of *HvNRT1.1*, *HvNRT2.1*, and *HvNRT1.5* in root tissue 22 days after application in field 1 (73% N fertilizer rate). Results are expressed as the relative log_2_ fold-change with respect to the *HvUBC9* gene expression level and benchmarked against untreated plants growing under 100% N fertilizer rate. The error bars represent SE and means followed by asterisk within the same gene and field indicate significant differences between control and the PSI-362 treatment based on *t*-test at *p* ≤ 0.05. Number of biological replicates (*n* = 3).

The PSI-362 coated fertilizer was applied at mid tillering stage (GS22-27), just before the stem elongation phase (GS30-39), which is the most important timing on spring barley for N uptake (Teagasc, 2015). In order to assess the long term impact of the biostimulant on N uptake and assimilation parameters, a metabolic analysis of shoot samples collected 22 days after application was performed. N uptake in control and treated plants at stage GS30-31 was evaluated through the measurement of shoot nitrate content. Plants treated with PSI-362 coated fertilizer significantly increased this parameter in field 1 (+17.87%, *p* = 0.046) and field 2 (+72.23%, *p* ≤ 0.001) compared to control plants growing under 100% N rate (Table 2). To provide some insights on the effect of PSI-362 coated fertilizer on N uptake mechanisms, the relative gene expression levels of three major nitrate transporters, namely *NRT1.1* and *NRT2.1*, both expressed in epidermal and cortical cells, and *NRT1.5*, expressed in root pericycle cells were measured (Vidal et al., 2020). The root tissue of barley plants at stage GS30-31 was collected from plants of field 1, which had a similar N fertilizer program as the field trials performed between 2016 and 2018. The application of PSI-362 coated fertilizer caused a significant upregulation within *NRT1.1*, *NRT2.1*, and *NRT1.5* expression levels by 1.77-, 1.72-, and 1.21-fold with respect to the 100% N control (*p* ≤ 0.001), suggesting increased root capacity for nitrate uptake and transport in barley crop under field conditions (Figure 4).

**TABLE 2:** Effect of PSI-362 coated CAN + S fertilizer on N forms (nitrate and ammonia), free amino acids, soluble protein, and photosynthetic pigments in barley shoot tissue (cv. Gangway) at 22 days after application.

Metabolite^1^	Field 1		Field 2	
	Control 100% N	PSI-362 coated 73% N	Control 100% N	PSI-362 coated 88% N
Nitrate (mg⋅g^–1^ DW)	12.72 ± 0.45	14.99 ± 0.66*	14.15 ± 1.57	24.37 ± 0.39***
Ammonia (mg⋅g^–1^ DW)	0.16 ± 0.01	0.13 ± 0.01*	0.21 ± 0.01	0.16 ± 0.01*
Free total amino acids (mg⋅g^–1^ DW)	36.40 ± 1.62	44.86 ± 1.21*	36.80 ± 2.42	45.16 ± 1.69*
Glutamate (mg⋅g^–1^ DW)	7.29 ± 0.29	8.68 ± 0.26**	6.91 ± 0.35	10.15 ± 0.55***
Glutamine (mg⋅g^–1^ DW)	1.38 ± 0.04	1.78 ± 0.05***	1.02 ± 0.08	2.58 ± 0.12***
Aspartate (mg⋅g^–1^ DW)	2.26 ± 0.10	2.89 ± 0.14**	1.36 ± 0.09	2.89 ± 0.14***
Asparagine (mg⋅g^–1^ DW)	0.94 ± 0.19	1.11 ± 0.18	0.43 ± 0.05	2.30 ± 0.13***
Proline (mg⋅g^–1^ DW)	0.66 ± 0.01	0.77 ± 0.01*	0.47 ± 0.02	0.57 ± 0.02*
Alanine (mg⋅g^–1^ DW)	1.25 ± 0.03	1.41 ± 0.04*	1.38 ± 0.11	1.59 ± 0.19
Serine (mg⋅g^–1^ DW)	0.96 ± 0.05	0.98 ± 0.04	0.74 ± 0.02	0.87 ± 0.03*
Glycine (mg⋅g^–1^ DW)	0.11 ± 0.01	0.12 ± 0.01	0.16 ± 0.01	0.26 ± 0.01***
Methionine (mg⋅g^–1^ DW)	0.20 ± 0.01	0.28 ± 0.03*	0.25 ± 0.01	0.27 ± 0.01
Lysine (mg⋅g^–1^ DW)	0.45 ± 0.02	0.50 ± 0.02	0.40 ± 0.01	0.47 ± 0.03*
Threonine (mg⋅g^–1^ DW)	0.33 ± 0.02	0.37 ± 0.02	0.27 ± 0.02	0.48 ± 0.05***
Soluble protein (mg⋅g^–1^ DW)	115.61 ± 4.34	144.35 ± 6.10***	136.04 ± 4.12	165.75 ± 4.58***
Total chlorophyll (a + b) (mg⋅g^–1^ DW)	8.63 ± 0.32	11.75 ± 0.22**	6.55 ± 0.23	10.17 ± 0.20***
Carotenoids (mg⋅g^–1^ DW)	1.79 ± 0.08	2.34 ± 0.14*	1.52 ± 0.05	2.10 ± 0.03***

^1^Data are the means ± SE. Number of biological replicates (n ≥ 4). Difference between control and treated sample within the same field was significant at *p ≤ 0.05, **p ≤ 0.01, and ***p ≤ 0.001, respectively (t-test).

### Effect of PSI-362 Coated Fertilizer on N Assimilation Metabolic Parameters

In order to assess the impact of PSI-362 coated fertilizer 22 days after application on well-known N assimilation parameters, a metabolic analysis of shoot samples from control and treated plants at early stem elongation stage (GS31-32) was performed. While reduced N fertilizer levels decreased shoot ammonium content in treated plants from field 1 (−15.56%, *p* = 0.044) and field 2 (−20.89%, *p* = 0.015), an opposite trend was observed in the content of total free amino acids. The effect of PSI-362 coated fertilizer on this N-assimilation parameter was consistent. Treated plants from field 1 and field 2 growing under 73 and 88% N fertilization levels, respectively, had significantly increased total free amino acid content in shoot tissue by 23.25% (*p* = 0.021) and 22.71% (*p* = 0.050) compared to the untreated shoots (Table 2). Glutamate was the major component of the free amino acids pool, together with glutamine, aspartate, asparagine, alanine, and serine. PSI-362 coated fertilizer strongly affected glutamate, increasing it to 18.95% (*p* = 0.006) and 46.84% (*p* ≤ 0.001) of the control values in shoot tissue of field 1 and field 2, respectively. This biostimulant treatment had a similar effect on glutamine, another key N metabolite that has an important role as amino group donor to form other amino acids, with a significant increase of 29.33 and 153.30% (*p* ≤ 0.001) in PSI-362 treated plants. Aspartate increased by 28.04% (*p* = 0.005) and 112.87% (*p* ≤ 0.001) in treated shoots from field 1 and field 2, respectively; while asparagine only showed a significant 4.3-fold increase (*p* ≤ 0.001) in treated plants from field 2. The results in Table 2 also demonstrated that in barley plants treated with PSI-362 coated fertilizer the endogenous proline content increased significantly in both fields compared to 100% N control (+16.41–20.16%; *p* ≤ 0.05). This same stimulating effect by coated PSI-362 fertilizer under reduced N rate was also observed for alanine (+12.83–14.61%), serine (+2.94–18.12%), glycine (+7.70–55.15%), and essential amino acids such as methionine (+8.07−37.90%), lysine (+12.19−19.20%), and threonine (+14.27–80.48%) content.

The ability of PSI-362 coated fertilizer to influence N assimilation early in the stem elongation stage was also evaluated through the measurement of soluble protein and photosynthetic pigments content in shoot tissue (Table 2). Higher protein content in shoot tissue relative to 100% N control were observed for barley plants fertilized with PSI-362 coated CAN + S at 73% N (+24.86, *p* ≤ 0.001) and 88% N (+21.84, *p* ≤ 0.001) rate. Accumulation of both chlorophylls (a + b) was similarly affected in treated plants, increasing by 36.24% (*p* = 0.003) and 30.28% (*p* = 0.050) in field 1 and field 2, respectively. Finally, this ANE biostimulant also induced a statistically significant accumulation of carotenoids in the shoots of barley plants growing with reduced N (+40.50–60.92%).

### Effect of PSI-362 Coated Fertilizer on NR and GS Activity

**FIGURE 5:**
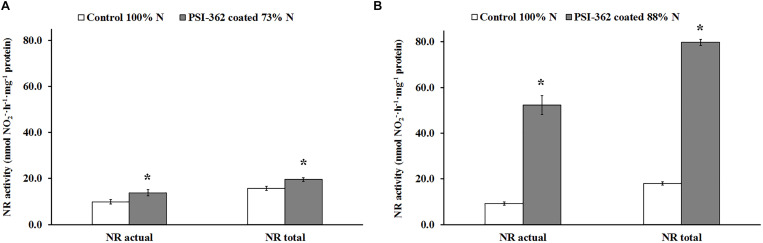
NR activity in spring barley shoot tissue (cv. Gangway). The effect of PSI-362 coated CAN + S fertilizer on actual NR (in presence of MgCl_2_) and total NR (in absence of MgCl_2_) activity was evaluated in shoot tissue 22 days after application. **(A)** Barley crop grown in the field 1 under 73% N recommended fertilizer rate; **(B)** barley crop grown in the field 2 under 88% N recommended fertilizer rate. Results in treated plants were benchmarked against untreated plants growing under 100% N fertilizer rate. Means followed by asterisk within the same NR activity type and field indicate significant differences between control and the PSI-362 treatment based on *t*-test at *p* ≤ 0.05. Number of biological replicates (*n* ≥ 4).

Total NR activity in barley shoots of control plants growing under 100% N rate was very similar for both field 1 and field 2 (15.69 and 17.94 nmol NO_2_^–^⋅h^–1^⋅mg^–1^ protein, respectively) (Figure 5). As expected, actual NR activity in presence of MgCl_2_ was lower and the calculated activation state for untreated plants ranged between 60.91% (field 1) and 50.92% (field 2). While the application of PSI-362 coated fertilizer led to a significant increase in NR activity in shoot tissue of plants at stage GS31-32, the magnitude of this variation differed between field 1 and field 2, being directly correlated to nitrate content measured in the same tissue (Table 2). Treated plants growing under 73% N increased their total NR activity in shoot tissue by 24.82% (*p* = 0.019) compared to control (Figure 5A). However, this activity was 4.7-fold higher in shoots of treated plants growing in field 2 (Figure 5B). Interestingly, the NR activation state also increased significantly with PSI-362 coated fertilizer treatment (*p* ≤ 0.001), reaching values of 70.53 and 65.63% for plants from field 1 and field 2, respectively.

**FIGURE 6:**
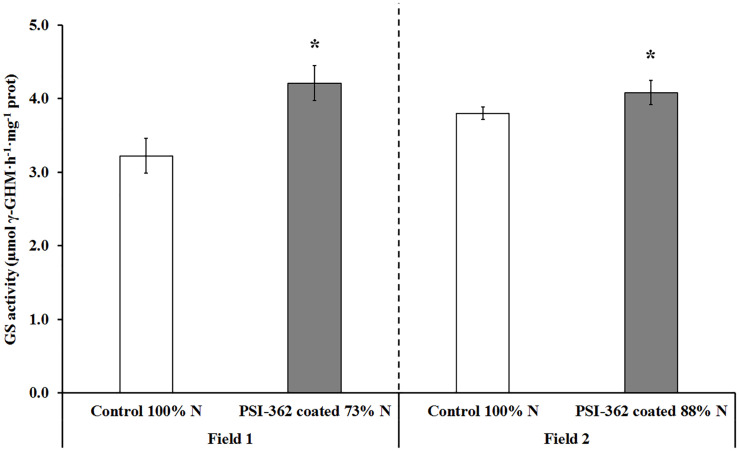
GS activity in spring barley shoot tissue (cv. Gangway). The effect of PSI-362 coated CAN + S fertilizer on GS activity was evaluated in shoot tissue 22 days after application. **Field 1:** Barley crop grown in the field 1 under 73% N recommended fertilizer rate. **Field 2**: barley crop grown in the field 2 under 88% N recommended fertilizer rate. Results in treated plants were benchmarked against untreated plants growing under 100% N fertilizer rate. Means followed by asterisk within the same field indicate significant differences between control and the PSI-362 treatment based on *t*-test at *p* ≤ 0.05. Number of biological replicates (*n* ≥ 4).

GS activity was also assayed in shoots of barley plants 22 days after the second N fertilizer application. Similarly to NR activity, control plants growing under 100% N rate showed comparable GS activity values in field 1 and field 2 (3.22 and 3.80 μmol γ-GHM⋅h^–1^⋅mg^–1^ protein, respectively). GS activity was also significantly stimulated by PSI-362 coated fertilizer, increasing its γ-glutamyl transferase activity in plants from field 1 (+30.63%, *p* = 0.014) and field 2 (+7.39%, *p* = 0.035) (Figure 6).

### Effect of PSI-362 Coated Fertilizer on Plant and Grain Parameters at Harvest

The results presented in Table 3 indicate that the application of PSI-362 coated fertilizer in barley grown under reduced N fertilizer rate did not have a statistically significant effect on total plant biomass at harvest compared to control crop fertilized at 100% N rate. While a reduction of 27% N in field 1 was translated in a higher biomass in treated plants (+8.14%, *p* = 0.319), no significant difference was recorded in field 2 (−0.94%, *p* = 0.957). Barley plants from field 1 fertilized with PSI-362 coated CAN + S were characterized as having a significantly higher number of tillers at maturity stage (+23.30%, *p* = 0.030), but this effect was not observed in field 2 (Table 3). Interestingly, the application of PSI-362 coated fertilizer was characterized by a higher harvest index in both fields (11.31–18.37%), and this increase in dry matter partitioned to the grain was statistically significant for treated barley plants growing with 88% N (*p* = 0.044).

**TABLE 3:** Effect of PSI-362 coated CAN + S fertilizer on plant and grain derived parameters in barley crop (cv. Gangway) at harvest.

Parameter^1^	Field 1		Field 2	
	Control 100% N	PSI-362 coated 73% N	Control 100% N	PSI-362 coated 88% N
Plant biomass^2^ (g plant^–1^)	7.15 ± 0.04	7.74 ± 0.25	6.53 ± 0.73	6.47 ± 0.48
Number tillers per plant	3.17 ± 0.14	3.90 ± 0.17*	3.32 ± 0.13	3.19 ± 0.12
Harvest index (g⋅g^–1^)	0.57 ± 0.05	0.67 ± 0.04	0.53 ± 0.02	0.60 ± 0.01*
Grain yield^2^ (T⋅ha^–1^)	6.36 ± 0.15	6.60 ± 0.11*	9.93 ± 0.60	9.63 ± 0.28
TGW (g)	52.58 ± 0.28	52.49 ± 0.26	48.24 ± 0.80	50.02 ± 0.50
Grain protein concentration (%w/w)	8.36 ± 0.39	8.31 ± 0.13	9.60 ± 0.09	10.80 ± 0.01***
Grain P content (%w/w)	0.28 ± 0.02	0.28 ± 0.03	0.28 ± 0.02	0.26 ± 0.02
Grain K content (%w/w)	0.44 ± 0.02	0.42 ± 0.03	0.44 ± 0.04	0.43 ± 0.02

^1^Data are the means ± SE. Number of biological replicates (n ≥ 4). ^2^Plant biomass, grain yield and grain characterization were expressed at 20% moisture. Difference between control and treated sample within the same field was significant at *p ≤ 0.05, and ***p ≤ 0.001, respectively (t-test).

Harvested grain yield differences between control plants grown under 100% N and those treated with PSI-362 coated fertilizer at reduced N rate showed a similar trend to that observed in barley trials developed from 2016 to 2018: either these differences were significantly higher in treated plants growing under 73% N rate (+3.70%, *p* = 0.042) or PSI-362 coated fertilizer led to no statistically significant difference in grain yield compared to control in field 2 (*p* = 0.780). An opposite trend was observed for the thousand grain weight (TGW) and protein content in both fields. While there were minimal differences in both parameters between treated and control crop in field 1, a significant increase of grain protein content (+12.50%, *p* ≤ 0.001) and higher TGW (3.68%, *p* = 0.095) was observed in barley treated with PSI-362 fertilizer at 88% N rate compared to untreated crop grown under 100% N rate. Concerning other macronutrients such as P and K, there were no statistically significant differences in their content between grain harvested from treated and untreated plants (Table 3).

### Effect of PSI-362 Coated Fertilizer on NUE and Environmental Parameters

The process of conversion of N fertilizer into grain biomass was evaluated through the amount of grain yield harvested per amount of N supplied to plants (*NUE*_*grain*_). There was a consistent increase of this parameter for treated crop in field 1 (+41.83%, *p* ≤ 0.001) and field 2 (+9.13%, *p* = 0.225), which agrees with the observed results in previous trials with PSI-362 applied as foliar spray (Supplementary Table 7). Because *NUE*_*grain*_ is mathematically the product of *Nupeff*_*grain*_ × *Nuteff*_*grain*_, both second level traits were also calculated. The plant capacity to take up soil available N by the plant and allocate it to the grain was measured through *Nupeff*_*grain*_ trait. Overall, barley crop treated with PSI-362 coated fertilizer significantly enhanced this parameter in both field 1 (+40.95%, *p* ≤ 0.001) and field 2 (+21.92%, *p* = 0.011), being the main contribution to the observed variation between treated and control *NUE*_*grain*_ and confirming the ability of PSI-362 to recover N more efficiently (Table 4). However, we did not observe statistically significant differences between treated and untreated plants in the *Nuteff*_*grain*_, remaining similar in field 1 (+0.36%, *p* = 0.942) or decreasing in field 2 (−10.49%, *p* = 0.355). Therefore, it indicates that this trait was not substantially impacting on *NUE*_*grain*_ variation (Table 4).

**TABLE 4:** Effect of PSI-362 coated CAN + S fertilizer on NUE and environmentally derived parameters in barley crop (cv. Gangway) at harvest.

Parameter^1^	Field 1		Field 2	
	Control 100% N	PSI-362 coated 73% N	Control 100% N	PSI-362 coated 88% N
*NUE_*grain*_ (kg grain⋅kg*^–1^ N fertilizer)	45.25 ± 1.31	64.18 ± 1.06***	62.81 ± 4.89	68.54 ± 1.96
*Nupeff*_*grain*_ (kg N grain⋅kg^–1^ N fertilizer)	0.65 ± 0.06	0.91 ± 0.01***	1.04 ± 0.05	1.27 ± 0.01*
*Nuteff*_*grain*_ (kg grain⋅kg^–1^ N grain)	69.76 ± 2.26	69.97 ± 1.38	60.31 ± 4.70	53.98 ± 1.55
GHG emissions (tCO_2_e⋅ha^–1^)	2.23 ± 0.07	1.74 ± 0.05***	2.54 ± 0.19	2.31 ± 0.07

^1^Data are the means ± SE. Number of biological replicates (n ≥ 4). Difference between control and treated sample within the same field was significant at ^∗^p ≤ 0.05, and ***p ≤ 0.001, respectively (t-test).

Despite these differences on N uptake efficiency, there were no statistically significant differences in the soil total N between control and treated plots after harvesting the crop (Supplementary Tables 4, 5). In addition, there were also no statistically significant differences in the content of available P and K before sowing and after harvesting in either field 1 or field 2. A higher content of amino sugar determined through the Illinois test in the organic fraction of soils treated with PSI-362 coated fertilizer after harvesting in field 1 (+30.21%, *p* = 0.085) and field 2 (+20.83%, *p* = 0.096) was recorded.

Estimated GHG emission calculations show that the application of PSI-362 led to a reduction in GHG emissions per hectare of cultivated area. The carbon equivalent emission estimate decreased nearly 22% for the treated crop growing under 73% N in field 1 (−483.52 kgCO_2_e⋅ha^–1^), whereas this reduction was closer to 9% (−224.49 kgCO_2_e⋅ha^–1^) with the application of PSI-362 coated fertilizer at 88% N rate in field 2 (Table 4).

## Discussion

Nitrogen fertilizer application rates are currently unsustainable, both environmentally (e.g., N pollution of soil, water and GHG emissions) and economically (e.g., reduced grower margins). A recent data compilation from 230 studies has reported that cereal crops only take up between 36% and 42% of applied N fertilizer (Yan et al., 2020), confirming previous low NUE estimations for cereal production (Raun and Johnson, 1999; Omara et al., 2019). Therefore, enhancing NUE is a necessity for developing sustainable agricultural production to feed a growing world population. The ability of ANE biostimulants to influence N uptake and assimilation mechanisms have been previously investigated in controlled conditions in the model plant *A. thaliana* (Durand et al., 2003), and crop plants such as oilseed rape (Jannin et al., 2013; Billard et al., 2014) and durum wheat (Laurent et al., 2020). In this study, it has been demonstrated under field conditions that an engineered biostimulant derived from *Ascophyllum nodosum*, PSI-362, is capable of increasing *NUE*_*grain*_ in barley by 29.85–60.26% when compared to current standard grower practices without compromising yields. Two methods of application of the biostimulant to barley were evaluated; foliar spray and top dressing via biostimulant coated granular fertilizer, with the effectiveness of the biostimulant evident for both methods in pot and field trials. Along with several enhanced N assimilation markers, a key indicator of biostimulant performance was increased nitrate content in barley shoot tissue 22 days after N fertilizer application. Additional plant phenotype, metabolic, enzymatic, and genetic analysis was performed on top dressed PSI–362 coated CAN + S barley crops to further support its role in enhancing NUE.

### Impact of PSI-362 on Phenotypic and Yield Markers

Current knowledge shows that NUE is a highly complex trait derived from diverse molecular, metabolic, physiological, developmental, and environmental interactions over the entire life cycle of the crop. Field experiments using a randomized block trial design for the ANE PSI-362 applied by foliar spray at three critical growth stages of barley provided agronomically relevant results during three consecutive growing seasons. While tested varieties showed fluctuations on harvested grain yield on their response to PSI-362, the measured values were in the range expected for spring (5.6–7.3 t⋅ha^–1^) and winter (8.6–9.1 t⋅ha^–1^) barley in Ireland between 2016 and 2018 (CSO, 2019). Most importantly, the field experiments demonstrated that the foliar application of an engineered ANE biostimulant was able to maintain or increase grain yield in barley grown with 25% less N fertilizer.

In order to expand the applicability and facilitate the implementation of PSI-362 in standard grower programs, PSI-362 was co-formulated with a N mineral granular fertilizer (CAN + S). Granular CAN based fertilizer is an efficient source of N with both nitrate and ammonium forms to maximize plant growth (Hachiya and Sakakibara, 2017). This N fertilizer is widely used by growers in Western Europe (2.16 Mt N⋅year^–1^), representing 51% of total worldwide consumption in 2019 (IFASTAT, 2019). Synchronizing N fertilizer application with the crop demand is critical for increasing NUE and reducing fertilizer losses. The co-formulation of CAN + S and PSI-362 is in-keeping with the 4R nutrient framework for increasing crop NUE: right rate, right source, right timing, and right placement. Previous research has shown that the critical period to positively influence spring barley yield and grain quality is the tillering and early stem elongation growth stages (Křen et al., 2015). These growth stages are characterized by a significant increase of N uptake, dry matter accumulation and responsiveness to N fertilization in terms of further grain yield (Baethgen et al., 1995; McTaggart and Smith, 1995; Hauggaard-Nielsen et al., 1998; Chatskikh and Olesen, 2007). Therefore, increasing uptake with PSI-362 at this growth stage was targeted based on the observed benefits of foliar applied PSI-362 enhancing NUE. Application of PSI-362 directly to the soil at the same time as the main N fertilizer application at the mid tillering stage was anticipated to provide similar benefits by stimulating N uptake, transport and assimilation mechanisms from root and shoot tissues before the stem elongation growth stage, compensating for the decreased N rate. The pot experiments revealed that the same dose of PSI-362 delivered by either foliar spray or coating of CAN + S granules enhanced plant biomass and NUE in an equivalent way.

Environmental conditions during the trials in field 1 and field 2 were favorable for spring barley production. The average Irish grain yield from the 2019 season was 8.0 t⋅ha^–1^ (CSO, 2020), which was 11% higher than the average yield recorded for the period 2014–2018 (Teagasc, 2018). While variations below and above this average grain yield value from field 1 and field 2 were likely related to differences in the grower program, soil chemical characteristics or crops grown in the previous season, our current results demonstrated that a moderate (i.e., 11–27%) reduction in N rate, combined with a single application of PSI-362 coated fertilizer, can maintain or increase grain yield, confirming the previous trend observed with three foliar applications of this biostimulant. Previous research about the effects of breeding on west European and Argentinean spring barley varieties from 1931 have demonstrated that overall improvements in grain yield can be achieved via different phenotypic routes (Abeledo et al., 2008; Bingham et al., 2012). Interestingly, this biostimulant treatment was able to improve important yield components such as plant biomass and harvest index, with no effect on grain size.

### PSI-362 Coated Fertilizer and N Uptake Mechanisms

The ability to take up and transport more nitrate from roots to shoots under reduced N fertilizer conditions has been observed as an adaptative response in wild barley genotypes to cope with low N field conditions (Quan et al., 2016). The top-dressing application of PSI-362 coated CAN + S to barley plants at mid-tillering stage (GS22-27) was also translated into a substantial stimulation of nitrate uptake and transport processes 22 days after application despite the reduction of N fertilizer rate, providing a significantly higher nitrate content in shoot tissue at stage GS30-31. These results were consistent with the proof of concept nitrate accumulation results in the model plant *Arabidopsis thaliana* 6 days after applying PSI-362 through the root under two different N levels. Although previous studies have highlighted the positive impact of ANEs on N uptake of oilseed rape and durum wheat growing under optimum nutrient conditions (Jannin et al., 2013; Billard et al., 2014; Laurent et al., 2020), no research to date has demonstrated the effect of ANEs on nitrate accumulation under reduced N fertilizer rate in real field conditions. PSI-362 ANE biostimulant did not possess any relevant N content to explain this nitrate accumulation effect in barley. The ability of specific biomolecules within commercial ANEs to induce plant growth and abiotic stress defense responses has been recently reviewed (Shukla et al., 2019; Goñi et al., 2020), with some of these studies reporting that ANEs can significantly dysregulate relevant genetic markers (Gonñi et al., 2016; Goñi et al., 2018; Shukla et al., 2018; Łangowski et al., 2019; Carmody et al., 2020). To elucidate PSI-362 mode of action at the molecular level, the expression of three nitrate plasma membrane carriers (NRT1.1, NRT1.5, and NRT2.1) was tested. A strong increase of expression of these genetic markers in roots of treated barley plants growing under 73% N rate was found, indicating that they could have a prominent role in facilitating N accumulation in shoot tissue. *NRT1.1* is an extensively studied gene that codes a dual affinity nitrate transporter and can also act as a nitrate transceptor. *NRT1.1* expression is known to be highly responsive to the amount of nitrate available in the environment as well as phosphorylation events under hormonal control (Lay-Pruitt and Takahashi, 2020). NRT2.1 is another crucial component controlling high affinity nitrate transport and predominantly localizes to the plasma membrane of root cells (Vidal et al., 2020). The long-distance nitrate transport between root and shoot is regulated by the gene *NRT1.5*, coding a low-affinity bidirectional nitrate transporter (Lin et al., 2008). Enhanced expression of *NRT1.5* gene in root tissue and increased nitrate transport from root to shoot through the xylem vascular tissues has been observed previously in a high NUE oilseed rape genotype (Han et al., 2016). Increased *NRT1.1* transceptor expression along with high-affinity transporter *NRT2.1* and long-distance transporter *NRT1.5* have implications for auxin biosynthesis and root architecture. Analysis of auxin signaling reporter lines combined with analysis of auxin biosynthesis and transport would be necessary to fully understand PSI-362 effect on plant development and physiology and what is the role of NRT1.1 as integrator. The observed nitrate accumulation in shoot tissue suggests that this biostimulant application could also increase the nitrate transport capacity in multiple ways. For example, through phosphorylation/dephosphorylation events controlled by CIPK23 kinase (Vidal et al., 2020), which can switch NRT transporters to high-affinity mode and facilitate the nutrients flow. Although it is tempting to speculate, nitrate uptake is a complex process, therefore detailed analysis of short- and long-term effects on root and shoot transcriptome need to be performed to fully comprehend the intricacy of this ANE biostimulant effect on NUE.

### PSI-362 Coated Fertilizer and Enzymes Involved in N Assimilation Mechanisms

Nitrate assimilation occurs first via NR enzyme to nitrite and then via nitrite reductase (NiR) to ammonium (Tischner, 2000; Masclaux-Daubresse et al., 2010). PSI-362 coated fertilizer did enhance nitrate assimilation at stage GS30-31 through a direct effect on increased NR enzymatic activity in shoot tissue, this change being positively correlated to nitrate accumulation. Gene expression, protein translation and enzymatic activity of NR is positively regulated by nitrate, light, and carbohydrates (Kaiser et al., 2002; Lillo, 2008; Yanagisawa, 2014; Huarancca Reyes et al., 2018). However, this regulation does not necessarily happen simultaneously at these three levels (Carillo et al., 2005). NR enzyme from PSI-362 treated plants was also found to be in a higher constitutively active state compared to untreated plants, regardless of N reduction rate in field 1 and field 2. This enhancement of the NR activation state indicates that PSI-362 may modulate the phosphorylation state of this enzyme 22 days after application, improving shoot nitrate reducing capacities under more adverse growth conditions. At the post-translational level, NR undergoes a partial kinase-dependent reversible inhibition, due to a phosphorylation of a serine residue in hinge 1 followed by a binding to 14-3-3 proteins in presence of divalent cations or polyamines. Therefore, NR activity in the presence of MgCl_2_ usually reflects the activity of dephosphorylated NR forms versus the activity of all NR forms in presence of EDTA (Athwal and Huber, 2002; Yanagisawa, 2014).

### PSI-362 Coated Fertilizer and N Assimilation to Amino Acids

The ammonium produced from reduced nitrate is fixed into the amino acid glutamine through the enzyme GS, constituting the first step in the biosynthesis of organic N compounds and serving as the cornerstone of N assimilation along with GOGAT enzyme (Hirel and Krapp, 2020). The current results showed that GS activity was significantly higher in plants treated with PSI-362 coated fertilizer, enhancing the nitrate assimilation pathway under reduced N rate, which in turn was associated to a lower content of ammonium and an accumulation of glutamine, both substrate and end-product of this enzyme. While GS has been proposed as an interesting target for genetic modification for NUE, attempts to overexpress GS have yielded inconsistent results (James et al., 2018). A recent cisgenic strategy to increase expression of native cytosolic GS1 in barley provided an enhanced enzymatic activity and did increase grain yield and NUE (Gao et al., 2019). Moreover, correlation studies performed in several wheat varieties suggested the presence of a strong relationship between GS activity and different N metabolic markers such as amino acids, soluble protein, and chlorophyll (Kichey et al., 2006, 2007). The increased glutamine assimilation occurred in a coordinated manner with the accumulation of asparagine, glutamate and other amino acids derived from glutamate through transamination reactions such as alanine, aspartate or proline, or essential amino acids derived from aspartate (e.g., methionine, lysine, and threonine) (Pratelli and Pilot, 2014). Therefore, it seems that PSI-362 coated fertilizer had a positive influence on stimulating barley shoots ability to acquire inorganic N and assimilate it into glutamine, favoring other amino acid biosynthetic processes simultaneously.

### PSI-362 Coated Fertilizer and Additional Plant Metabolic Parameters

As recently reviewed by Fernie et al. (2020), any attempt to improve crop production and plant NUE through modification of N transport and metabolism pathways will only be effective if all processes are synchronized because unbalanced changes in internal N metabolite pools can generate end-product inhibition or substrate limitations of the respective amino acid synthesis pathways. Thus, the significant accumulation of total free amino acids in barley plants treated with PSI-362 coated fertilizer was also linked to a higher content of macromolecules derived from them such as soluble proteins or chlorophylls. An increased soluble protein content in barley shoot may be associated to a stimulated carbon fixation capacity because the majority of leaf soluble protein content in leaves of C3 plants is present as ribulose-1-5 bis-phosphate carboxylase-oxygenase (Rubisco), the key enzyme of the Calvin cycle (Feller et al., 2008). Together with this factor, the higher photosynthetic pigments content observed in shoot tissue could support this improvement of canopy photosynthesis at stage GS30-31 according to the model established by Cabrera-Bosquet et al. (2009). Overall, stimulating N uptake and assimilation mechanisms before the reproductive stage could facilitate subsequent N absorption during the grain filling process thereby compensating any detrimental effect associated with lower N fertilizer rate. Previous studies in winter wheat and barley have demonstrated that grain filling is mainly fueled with N remobilized from pre-anthesis biomass accumulation (Egle et al., 2008; Zhou et al., 2018).

### Impact of PSI-362 Coated Fertilizer on NUE, Soil, and Environmentally Derived Parameters

This research demonstrates that the intrinsically low barley *NUE*_*grain*_ can be enhanced consistently through the application of PSI-362, with this improvement being achieved through better *Nupeff*_*grain*_. Increasing *Nupeff*_*grain*_ trait as a way to further improve NUE has been suggested in previous breeding studies that evaluate barley and wheat varieties (Bingham et al., 2012; Thorwarth et al., 2018). However, unlike breeding programs that can take several years (Garnett et al., 2015), the application of PSI-362 coated fertilizer represents a more convenient, quicker, and profitable solution for growers to recover more N provided by reduced rates of N mineral fertilizer. The soil nitrogen analysis post-harvest suggests that soil N depletion is not occurring in the field trials performed with PSI-362. This finding is in agreement with previous observations over a wide range of N application, with similar amounts of residual N remaining in the soil after harvesting treated and untreated crops (Makowski et al., 1999; Bingham et al., 2012; Venterea et al., 2016). GHG emissions from the use of N mineral fertilizers are usually split into two parts: emissions from the energy-intensive manufacture process (mainly CO_2_ and N_2_O) and the emission of N_2_O from the fertilized soil (AHDB, 2012). The estimated results determined through the inputs and outputs recorded in the trials developed with PSI-362 coated fertilizer revealed a significant reduction of carbon footprint compared with current practice. Assuming savings of 483 kgCO_2_e⋅ha^–1^ as obtained with PSI-362 coated fertilizer under 73% N rate, a reduction of 27.5 MtCO_2_e can be expected per season if this agronomic input is implemented in the whole EU cereal production area (56.9 MHa; FAOSTAT, 2019).

### Summary and Perspectives

The data presented demonstrates that an engineered biostimulant, PSI-362, derived from *Ascophyllum nodosum*, is capable of increasing NUE in barley under field conditions. The targeted application of PSI-362 as a coating on a granular N mineral fertilizer enhanced N uptake, transport and assimilation markers at phenotypic, metabolic, enzymatic and genetic levels in a coordinated manner. The efficient uptake of more nitrate by the crop when it needs it, results in enhanced NUE derived traits in harvested grain. These results support the agronomic use of this biostimulant with its effect delivered through a defined physiological mode of action that allows up to 27% reduction in N fertilizer usage while maintaining or increasing crop yield. The magnitude of the nitrogen reduction achieved with PSI-362 without compromising yield suggests it can have a role in delivering the European Union (EU) target of a 20% reduction in nitrogen use in agriculture. However, in order to expand the applicability and impact of PSI-362 within agriculture, additional data supporting its efficacy in other important crops (e.g., grass, wheat, maize, etc.) may be beneficial. Further investigation of the mode of action at a basic plant science level to understand the optimal conditions for efficacy will be important to facilitate adoption of this NUE biostimulant technology. The availability of biostimulant innovations in the market to enhanced NUE are going to be critical for achieving more profitable, sustainable and environmentally acceptable agricultural practices.

## Data Availability Statement

The original contributions presented in the study are included in the article/Supplementary Material, further inquiries can be directed to the corresponding author.

## Author Contributions

OG, ŁŁ, EF, PQ, and SO’C conceived and designed the experiments. OG, ŁŁ, EF, and PQ performed the experiments and analyzed the data. OG, ŁŁ, EF, and SO’C wrote the article. All authors reviewed and approved the article. All authors contributed to the article and approved the submitted version.

## Conflict of Interest

Brandon Bioscience manufactures PSI-362. Third party growers performed field trials and provided the yield results. This project was supported partly by Enterprise Ireland. The funder provided support in the form of consumables and salary for authors (ŁŁ, OG, and EF), but did not have any additional role in the study design, data collection and analysis, decision to publish, or preparation of the manuscript. ŁŁ, OG, EF, and SO’C are employed by Brandon Bioscience. The remaining author declares that the research was conducted in the absence of any commercial or financial relationships that could be construed as a potential conflict of interest.
