# Supplementary material for: Reducing Nitrogen Input in Barley Crops While Maintaining Yields Using an Engineered Biostimulant Derived From Ascophyllum nodosum to Enhance Nitrogen Use Efficiency
Source: Front Plant Sci. 2021 May 5;12:664682. doi: 10.3389/fpls.2021.664682 (PMC8132967; doi:10.3389/fpls.2021.664682)
Supplement: Supplementary file 1 [file Data_Sheet_1.docx]

Supplementary Material

# Supplementary Tables

**Table S1. Characteristics of barley varieties used for field trials (2016-2019)**

| **Variety name** | **Description** | **Pathogen resistance** | **TGW (g)** | **Recommended DAFM/AHDB^1^** |
| --- | --- | --- | --- | --- |
| **KWS Irina** | German high yielding spring malting barley | Very good resistance to mildew and net blotch. Moderate resistance to brown rust and *Rhynchosporium*. | 50.7 | From 2014 |
| **Mickle** | British high yielding spring with very good grain quality | Moderately susceptible to brown rust. Susceptible to mildew and net blotch. Good resistance to *Rhynchosporium*. | 50.1 | From 2013 |
| **Gangway** | A Danish spring variety with very high yield potential. Excellent grain quality. | Very good resistance to mildew and net blotch, with good resistance to brown rust and *Rhynchosporium*. | 49.5 | From 2018 |
| **KWS Tower** | A two-row feed winter variety with good lodging resistance. | Susceptible to net blotch, but resistant to the common strains of barley mosaic viruses | 54.5 | From 2014 |

^1^ DAFM (Department of Agriculture, Food and the Marine; Ireland); AHDB (The Agriculture and Horticulture Development Board; United Kingdom).

**Table S2. Grower program for spring barley field trials (2016-2018)**

| **Agronomical input** | **Growth stage** | **Amount** |
| --- | --- | --- |
| **Lime** | Before sowing | 300-400 kg·ha^-1^ |
| **Fertiliser (10-10-20)** | Before sowing | 500 kg·ha^-1^ |
| **Herbicide (fluroxypyr)** | Early tillering (GS20-21) | 100 g·ha^-1^ |
| **Herbicide (metsulfuron-methyl and tribenuron-methyl)** | Early tillering (GS20-21) | 2 g·ha^-1^ |
| **Fertiliser (CAN, 27-0-0)** | Mid-tillering (GS22-27) | 388 kg·ha^-1^ (100% N)  248 kg·ha^-1^ (75% N) |
| **Fungicide (azoxystrobin)** | Early stem elongation (GS31-32) | 250 g·ha^-1^ |
| **Fungicide (prothioconazole)** | Early stem elongation (GS31-32) | 125 g·ha^-1^ |

**Table S3. Grower program for winter barley field trials (2016-2018)**

| **Agronomical input** | **Growth stage** | **Amount** |
| --- | --- | --- |
| **Lime** | Before sowing | 300-400 kg·ha^-1^ |
| **Fertiliser (10-10-20)** | Before sowing | 500 kg·ha^-1^ |
| **Fungicide (azoxystrobin)** | Early tillering (GS20-21) | 250 g·ha^-1^ |
| **Herbicide (fluroxypyr)** | Early tillering (GS20-21) | 150 g·ha^-1^ |
| **Herbicide (metsulfuron-methyl and tribenuron-methyl)** | Early tillering (GS20-21) | 2 g·ha^-1^ |
| **Fertiliser (CAN, 27-0-0)** | Mid-tillering (GS22-27) | 222 kg·ha^-1^ (100% N)  167 kg·ha^-1^ (75% N) |
| **Herbicide (fluroxypyr)** | End tillering (GS27-29) | 30 g·ha^-1^ |
| **Fertiliser (CAN, 27-0-0)** | Early stem elongation (GS31-32) | 370 kg·ha^-1^ (100% N)  278 kg·ha^-1^ (75% N) |
| **Grass herbicide (diflufenican)** | Early stem elongation (GS31-32) | 125 g·ha^-1^ |
| **Plant growth regulator (ethephon)** | Mid stem elongation (GS32-35) | 480 g·ha^-1^ |
| **Fertiliser (CAN, 27-0-0)** | Late stem elongation (GS37-39) | 148 kg·ha^-1^ (100% N)  111 kg·ha^-1^ (75% N) |
| **Herbicide (fluroxypyr)** | Late stem elongation (GS37-39) | 150 g·ha^-1^ |
| **Fungicide (azoxystrobin)** | Late stem elongation (GS37-39) | 250 g·ha^-1^ |
| **Fungicide (fenpropimorph)** | First ear emergence (GS51-52) | 500 g·ha^-1^ |

**Table S4. Soil chemical properties for Field 1 (2019)**

| **Parameter^1^** | **Before sowing** | |  | **After harvesting** | |
| --- | --- | --- | --- | --- | --- |
|  | **Control 100% N** | **PSI-362 coated 73% N** |  | **Control 100% N** | **PSI-362 coated 73% N** |
| **Bulk density (g·cm^-3^)** | 1.21 ± 0.05 | 1.20 ± 0.07 |  | 1.23 ± 0.03 | 1.21 ± 0.04 |
| **pH** | 6.50 ± 0.30 | 6.55 ± 0.40 |  | 6.85 ± 0.45 | 7.20 ± 0.40 |
| **Organic matter (g·kg^-1^ DW)** | 39.60 ± 1.79 | 39.40 ± 1.80 |  | 49.50 ± 0.80 | 44.60 ± 0.80* |
| **Total N (g·kg DW^-1^)** | 1.70 ± 0.32 | 1.72 ± 0.31 |  | 1.90 ± 0.30 | 1.80 ± 0.20 |
| **Amino sugar (mg·kg^-1^ N)** | 299.0 ± 22.0 | 305.0 ± 18.0 |  | 117.5 ± 10.0 | 153.0 ± 12.0 |
| **Available P (mg·kg^-1^ DW)** | 13.92 ± 2.83 | 13.70 ± 2.91 |  | 11.90 ± 2.20 | 14.45 ± 3.40 |
| **Available K (mg·kg^-1^ DW)** | 181.5 ± 34.0 | 184.8 ± 30.7 |  | 274.5 ± 35.0 | 282.5 ± 37.0 |

^1^ Data are the means ± SE. Number of biological replicates (*n* = 3). *: difference between control and treated sample within the same field was significant at *p* ≤ 0.05, respectively (t-test).

**Table S5. Soil chemical properties for Field 2 (2019)**

| **Parameter^1^** | **Before sowing** | |  | **After harvesting** | |
| --- | --- | --- | --- | --- | --- |
|  | **Control 100% N** | **PSI-362 coated 88% N** |  | **Control 100% N** | **PSI-362 coated 88% N** |
| **Bulk density (g·cm^-3^)** | 1.14 ± 0.06 | 1.16 ± 0.03 |  | 1.15 ± 0.05 | 1.13 ± 0.06 |
| **pH** | 6.90 ± 0.20 | 6.90 ± 0.30 |  | 6.85 ± 0.10 | 6.75 ± 0.15 |
| **Organic matter (g·kg^-1^ DW)** | 90.80 ± 2.40 | 89.60 ± 3.70 |  | 74.40 ± 3.50 | 89.60 ± 3.70 |
| **Total N (g·kg DW^-1^)** | 2.65 ± 0.25 | 2.84 ± 0.28 |  | 2.84 ± 0.12 | 2.76 ± 0.25 |
| **Amino sugar (mg·kg^-1^ N)** | 439.0 ± 36.0 | 425.0 ± 28.0 |  | 168.0 ± 5.0 | 203.0 ± 15.4 |
| **Available P (mg·kg^-1^ DW)** | 73.50 ± 4.20 | 69.50 ± 1.20 |  | 75.95 ± 1.90 | 73.75 ± 2.40 |
| **Available K (mg·kg^-1^ DW)** | 304.5 ± 29.0 | 318.9 ± 25.7 |  | 364.6 ± 10.0 | 389.9 ± 25.7 |

^1^ Data are the means ± SE. Number of biological replicates (*n* = 3). No statistically significant differences between control and treated sample at *p* ≤ 0.05 were observed (t-test).

**Table S6. Grower program for barley field trials using coated PSI-362 (2019)**

| **Agronomical input** | **Field 1** | |  | **Field 2** | |
| --- | --- | --- | --- | --- | --- |
|  | **Growth stage** | **Amount** |  | **Growth stage** | **Amount** |
| **Fertiliser (10-10-20)** | Before sowing | 380 Kg·ha^-1^ |  |  |  |
| **Fertiliser (22-8-0+S+B)** |  |  |  | Before sowing | 375 Kg·ha^-1^ |
| **Fertiliser (0-0-40)** |  |  |  | Before sowing | 375 Kg·ha^-1^ |
| **Insecticide**  **(Lambda-cyhalothrin)** | Leaf Development (GS13-15) | 0.1 L·ha^-1^ |  | Leaf Development (GS13-15) | 0.05 L·ha^-1^ |
| **Herbicide**  **(fluroxypyr)** | Leaf Development (GS15-19) | 0.7 L·ha^-1^ |  |  |  |
| **Herbicide**  **(metsulfuron-methyl & thifensulfuron-methyl)** | Leaf Development (GS15-19) | 0.04 Kg·ha^-1^ |  |  |  |
| **Herbicide**  **(Tribenuron methyl & Thifensulfuron methyl)** |  |  |  | Leaf Development (GS15-19) | 30 g·ha^-1^ |
| **Herbicide**  **(Fluroxypyr-meptyl, clopyralid & florasulam)** |  |  |  | Leaf Development (GS15-19) | 0.8 L·ha^-1^ |
| **Micro elements (Mn)** | Leaf Development (GS15-19) | 150 g·ha^-1^ |  | Leaf Development (GS15-19) | 150 g·ha^-1^ |
| **Fertiliser (CAN+S)** | Mid-tillering (GS22-27) | 380 kg·ha^-1^ (100% N)  240 kg·ha^-1^ (73% N) |  | Mid-tillering (GS22-27) | 280 kg·ha^-1^ (100% N)  215 kg·ha^-1^ (88% N) |
| **Plant growth regulator (chlormequat chloride)** | Mid-tillering (GS22-27) | 1.0 L·ha^-1^ |  |  |  |
| **Fungicide (bixafen & prothioconazole)** | Mid-tillering (GS 22-27) | 0.4 L·ha^-1^ |  | Mid-tillering (GS22-27) | 0.5 L·ha^-1^ |
| **Fungicide (Epoxiconazole, Fluxapyroxad & Pyraclostrobin)** |  |  |  | Mid-tillering (GS22-27) | 1.5 L·ha^-1^ |
| **Micro elements (Mn)** | Early stem elongation (GS31-32) | 150 g·ha^-1^ |  | Early stem elongation (GS31-32) | 150 g·ha^-1^ |
| **Plant Growth Regulator (Prohexadione & Trinexapac ethyl)** |  |  |  | Stem Elongation (GS30-39) | 0.3 L·ha^-1^ |
| **Fungicide (epoxiconazole, fluxapyroxad & pyraclostrobin)** | Stem Elongation (GS30-39) | 1.8 L·ha^-1^ |  |  |  |
| **Fungicide (chlorothalonil)** | Stem Elongation (GS30-39) | 1.0 L·ha^-1^ |  | Stem Elongation (GS30-39) | 1.0 L·ha^-1^ |
| **Fungicide (Prothioconazole)** |  |  |  | Stem Elongation (GS30-39) | 0.4 L·ha^-1^ |

**Table S7. Primers used for RT-qPCR analysis in barley.**

| **Primer** | **Sequence (5’-3’)** |
| --- | --- |
|  |  |
| **HvNRT1.1_FW, *HORVU7Hr1G071600*** | CCGGTATAGGGACTTGCACC |
| **HvNRT1.1_REV, *HORVU7Hr1G071600*** | CTTCTCGGTGCTGTTGGACT |
| **HvNRT2.1_FW, *HORVU6Hr1G005590.1*** | GCCCTTCGTAGCGTGGC |
| **HvNRT2.1_REV, *HORVU6Hr1G005590.1*** | CTTGCCCCATGGTGAGTACC |
| **HvNRT1.5_FW, *HORVU6Hr1G070450.3*** | CAACGTGGGCTCCATCTTCT |
| **HvNRT1.5_REV, *HORVU6Hr1G070450.3*** | GAGACCCAGAAGCCCATGAC |
| **HvUBC9_FW, *HORVU5Hr1G088270*** | CAACGTGGGCTCCATCTTCT |
| **HvUBC9_REV, *HORVU5Hr1G088270*** | GAGACCCAGAAGCCCATGAC |

**Table S8. Effect of foliar PSI-362 treatment on grain NUE of spring and winter barley varieties growing under 75% N fertiliser rate in 3 consecutive seasons (2016-2018).**

| **Season** | **Barley Variety (Spring/Winter)** | **NUE (kg grain·kg^-1^ N fertiliser)** | |
| --- | --- | --- | --- |
|  |  | **Control 100% N** | **Foliar PSI-362 75% N** |
| **2016** | KWS Irina (Spring) | 39.13 ± 1.42 | 62.72 ± 1.60*** |
| **2016/2017** | KWS Tower (Winter) | 31.84 ± 0.72 | 41.49 ± 1.01** |
| **2017** | KWS Irina (Spring) | 36.60 ± 0.49 | 52.53 ± 0.24** |
| **2017/2018** | KWS Tower (Winter) | 42.38 ± 0.08 | 58.69 ± 0.25*** |
| **2018** | Mickle (Spring) | 32.28 ± 1.45 | 41.92 ± 1.24** |

^1^ Data are the means ± SE. Number of biological replicates (*n* ≥ 4). **, ***: Difference between control and treated sample within the same field was significant at *p* ≤ 0.01, and *p* ≤ 0.001, respectively (t-test).
